# Defining the presymptomatic phase of frontotemporal dementia

**DOI:** 10.1097/WCO.0000000000001174

**Published:** 2023-06-23

**Authors:** Lucy L. Russell, Jonathan D. Rohrer

**Affiliations:** Dementia Research Centre, UCL Queen Square Institute of Neurology, University College London, London, UK

**Keywords:** C9orf72, frontotemporal dementia, primary progressive aphasia, progranulin, tau

## Abstract

Frontotemporal dementia (FTD) is a clinically, pathologically and genetically heterogeneous disorder. Whilst disease modifying therapy trials are mostly focused on the symptomatic phase, future studies will move earlier in the disease aiming to prevent symptom onset. This review summarizes the recent work to better understand this presymptomatic period.

**Recent findings:**

The presymptomatic phase can be split into preclinical and prodromal stages. The onset of the preclinical phase is defined by the first presence of pathological inclusions of tau, TDP-43 or fused in sarcoma in the brain. Definitive biomarkers of these pathologies do not yet exist for FTD. The prodromal phase is defined by the onset of mild symptoms. Recent work has highlighted the wide phenotypic spectrum that occurs, with the concept of mild cognitive ± behavioural ± motor impairment (MCBMI) being put forward, and additions to scales such as the CDR plus NACC FTLD now incorporating neuropsychiatric and motor symptoms.

**Summary:**

It will be important to better characterize the presymptomatic period moving forward and develop robust biomarkers that can be used both for stratification and outcome measures in prevention trials. The work of the FTD Prevention Initiative aims to facilitate this by bringing together data from natural history studies across the world.

## INTRODUCTION

Frontotemporal dementia (FTD) is a complex and heterogeneous neurodegenerative disorder with multiple different clinical phenotypes and pathological causes [1]. It is probably the most common cause of dementia in those under the age of 60 and like the other degenerative dementias there are currently no curative therapies. Clinically, the most common syndromes are behavioural variant FTD (bvFTD) in which personality and social cognition are affected and primary progressive aphasia (PPA) where language deficits occur. However, motor impairment is also seen in FTD, often manifesting as one of the atypical parkinsonian disorders, progressive supranuclear palsy (PSP) or corticobasal syndrome (CBS), or as amyotrophic lateral sclerosis (ALS). As well as clinical heterogeneity, the underlying neuropathology of these conditions is diverse, usually being associated with inclusions containing tau, TDP-43 or fused in sarcoma (FUS), although each of these can be divided further into multiple subtypes. Additionally, whilst around two thirds of people have a sporadic form, around one third have an autosomal dominant genetic cause with mutations in the chromosome 9 open reading frame 72 (*C9orf72*), progranulin (*GRN*) and microtubule associated protein tau (*MAPT*) genes being the most common.

Whilst much has been understood about the symptomatic period of FTD, less had been studied about the presymptomatic phase until relatively recently. For sporadic disease, the rarity of FTD makes prospective studies of this phase difficult, even within the context of large scale healthy aging cohorts. However, the genetic form allows a window into the premanifest stage by the study of at-risk individuals, who as a first-degree relative of a genetic mutation carrier, have a 50% chance that they too will carry the mutation. Early reports focused on single cases or small case series but within the last 10 years a number of large observational cohort studies of genetic FTD families have sprung up around the world including the GENetic FTD Initiative (GENFI) in Europe and Canada (www.genfi.org) and the ARTFL-LEFFTDS Longitudinal Frontotemporal Lobar Degeneration (ALLFTD) study in the US (www.allftd.org). These studies have come together under the FTD Prevention Initiative (FPI: www.thefpi.org) and of late have been joined by other cohorts in South America, Asia and Australasia to further understand the natural history of FTD.

The benefits of understanding the presymptomatic phase of FTD are multifold. Firstly, it allows a better understanding of the sequence of pathophysiological changes that occur in each type of FTD that in turn may inform underlying disease mechanisms and drug development. Secondly, it may lead to better prediction of symptom onset and likely progression of disease in at-risk individuals – at present, it is not possible to predict when people will develop symptoms, which phenotype they will have, or how fast their disease will proceed. Thirdly, it may allow a therapeutic window during which minimal neuronal loss has occurred and treatment may have the best chance of succeeding.

This review outlines the current thinking on the presymptomatic phase of FTD (including terminology, Table 1) as well as the outstanding issues that remain to be resolved (Tables 2 and 3). 

**Box 1:**
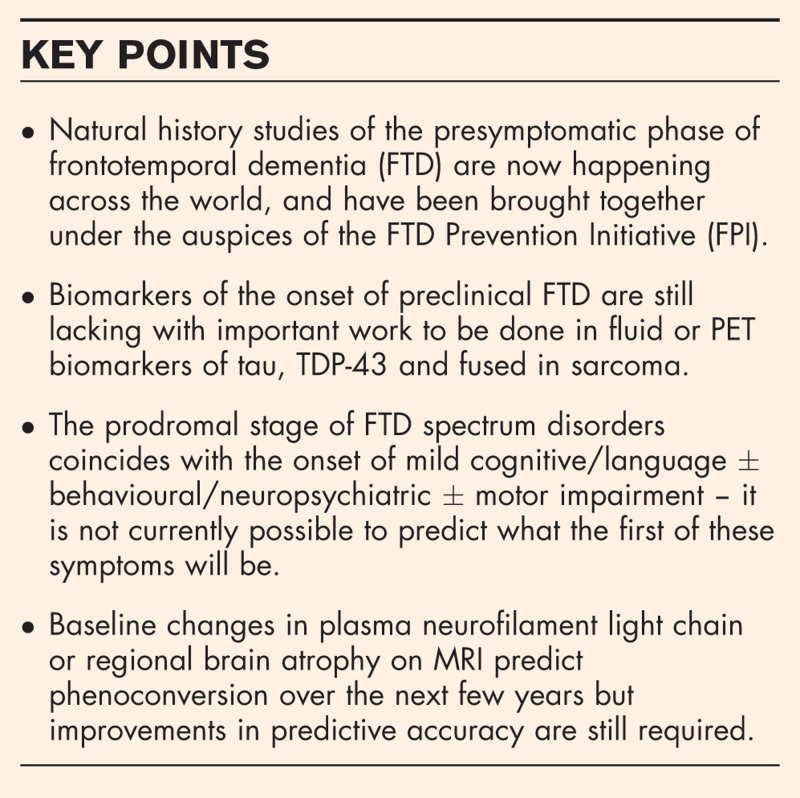
no caption available

**Table 1:** Terminology

**FIGURE 1:**
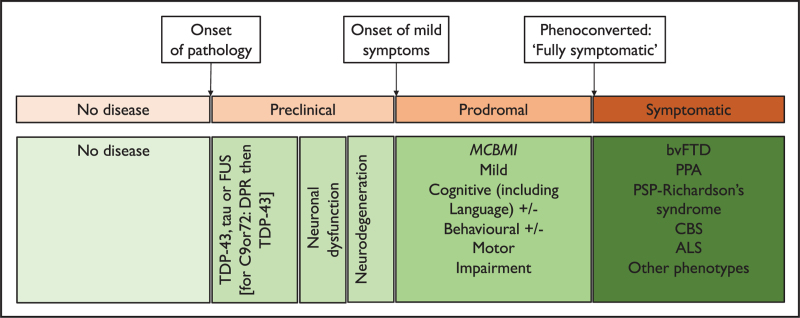
Stages of frontotemporal dementia.

• The presymptomatic phase can be broken down into a number of different stages (Fig. 1):
o Firstly, a no disease stage where there are no biological changes or clinical symptoms of the disease.
o Secondly, a preclinical stage where biological changes start to occur with biochemical abnormalities leading to deposition of abnormal proteins followed by neuronal dysfunction and neurodegeneration. However, no clinical symptoms are present at this stage.
o Lastly, there is the prodromal stage where clinical symptoms start to emerge but do not fulfil criteria for a formal diagnosis of frontotemporal dementia.
o Following the prodromal period is the symptomatic stage where the individual meets diagnostic criteria. The transition between the prodromal and symptomatic stages is referred to as phenoconversion.
• Several efforts have been made to better clarify the prodromal stage as has been done in the Alzheimer's disease field with MCI or mild cognitive impairment. However, the multiplicity of potential symptoms in FTD prevents a simple analogous name to be applied such as mild behavioural impairment. Not only is it not possible to predict (for genetic FTD) which phenotype an individual will develop, but people can develop multiple early symptoms concurrently. This has led to the development of the term MCBMI or mild cognitive and/or behavioural and/or motor impairment to encompass the whole spectrum of symptoms that might be present.

FTD, frontotemporal dementia; MCBMI, mild cognitive ± behavioural ± motor impairment.

**Table 2:** Outstanding questions regarding the presymptomatic phase of frontotemporal dementia (adapted from [32])

1. How do we define the onset of preclinical disease? *Whilst we now have a sensitive marker of DPRs in C9orf72-related disease, markers of TDP-43, tau and FUS need to be developed.*
2. How do we define further stages within preclinical disease i.e. neuronal dysfunction and neurodegeneration? *FDG-PET and potentially ASL-MRI and synaptic PET imaging may provide the earliest measure of neuronal dysfunction whilst structural MR imaging and plasma/CSF neurofilament light chain are promising measures of neurodegeneration.*
3. Is there a ‘no disease’ phase in genetic FTD preceding the onset of preclinical disease? *What is the earliest time point that pathological changes can be seen? Future studies of children with pathogenic mutations will be helpful to understand this once better measures of underlying pathology are developed.*
4. How do we define onset of prodromal disease? *Some deficits are more amenable to objective measurement e.g. cognitive or motor deficits but subtle deficits require more sensitive measures, and less objective measures such as behavioural changes require more thought about how best to measure earliest change.*
5. How may we best assess MCMBI due to FTD? *New scales and measures are required to identify the full spectrum of deficits seen in FTD.*
6. How do we include the prodromal neuropsychiatric features (particularly of *C9orf72*) within this framework? *This remains problematic, and a more nuanced assessment and understanding of changes in personality or changes suggestive of an autistic spectrum disorder or schizotypy, in childhood and early adulthood is required.*
7. How do we include mild features of parkinsonism or motor neuron disease within this scheme? *Much can be learned from related fields in the detection of early motor deficits including the use of digital measures or wearables.*
8. How do we define phenoconversion? *A grey zone exists as people move from a prodromal period to a fully symptomatic stage. This is made more complex by standard concepts of dementia that rely on the impairment of activities of daily living which are less relevant in the FTD syndromes (particularly in those with language syndromes). Definitive measures that can predict phenoconversion with high specificity and sensitivity need to be identified.*

FTD, frontotemporal dementia.

**Table 3:** Issues identified by presymptomatic members of families carrying a genetic mutation causative of FTD

• How often should assessment occur during the presymptomatic phase? During the early part of the presymptomatic phase many family members report anxiety about attending annual research visits. Example quote: ‘*Whilst I want to help with research, the research visit reminds me that I might develop this condition, whereas I can forget about it for the other 364 days of the year.’* In contrast, some family members, particularly as they get older, want to be monitored more regularly. Example quote: ‘*I would ideally have more regular tests done, perhaps every few months, to identify when things are starting. I could then do something about it such as join a trial.’*
• How do we best measure the potential onset of symptoms in the presymptomatic phase? Presymptomatic family members have concerns about relying on an informant report which is exclusionary of the at-risk person. It risks creating unwanted problems between that at-risk person and their partner. Example quote: *‘I worry [that my partner's report] might reflect something I was not aware of, or overemphasize isolated occurrences’.*
• What symptoms are most distressing to people and important to quality of life? Presymptomatic family members report concerns about language and cognitive impairment much more so than behavioural change. In contrast, early behavioural change is more distressing to the care partner. However, such symptoms change quite late in the presymptomatic period (by definition prodromally), and therefore for many people the most distressing symptom and the one that affects quality of life the most presymptomatically is a change in mood. Whilst some people can be majorly depressed or anxious, for many there is a harder to identify (and measure) chronic and longstanding effect on well being. This may be triggered by the ‘biographical disruption’ to their life when they find out they are at-risk of developing FTD.
• How do we assess whether people are likely to phenoconvert in the near future? Whilst imaging changes are seen in PET and MR many years before onset, on an individual basis it can be difficult to predict the time to symptom onset. The recent identification of NfL as a potential predictor of symptom onset over the following years has led to its use in trials as a stratification measure. The experience of presymptomatic family members who have had NfL measured has been mixed – whilst some have appreciated more clarity over what is happening, for others it has created more anxiety, particularly when they do not consider themselves to be near symptom onset. Example quote: *‘I am glad to be involved in a trial now but I wish I had not had to find out my [NfL] level – I am now continuously on the lookout for symptoms.’*

FTD, frontotemporal dementia.

## PRECLINICAL STAGE

### Onset of pathology

**FIGURE 2:**
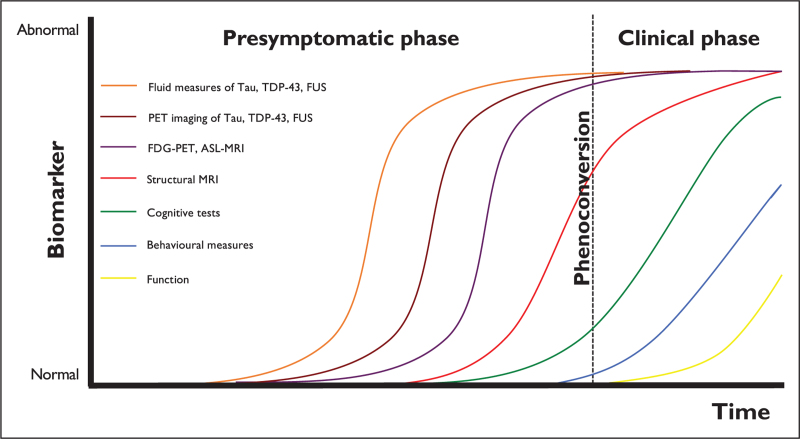
Theoretical biomarker changes during the presymptomatic and clinical phases of frontotemporal dementia.

For the majority of the different forms of FTD the onset of the preclinical stage is defined by the accumulation of tau, TDP-43 or FUS inclusions in the brain (Figs. 1 and 2). Unfortunately, there are currently no ways of definitively measuring these changes during life, with neither fluid measures within blood or cerebrospinal fluid (CSF) or positron emission tomography (PET) yet to produce a robust biomarker of underlying FTD pathology.

The most work has been done in tau, with CSF, and more recently blood, measures of phospho-tau accurately distinguishing Alzheimer's disease (AD) from controls and other degenerative disorders. However, these biomarkers do not identify the primary tauopathies [2], nor do the multiple different length tau fragments that have been investigated across multiple studies [3–5]. A potential CSF marker of a specific tau pathology that has been very recently studied is a 4-repeat isoform-specific tau species from the microtubule-binding region (MTBR-tau) – an initial study shows a decrease in people with corticobasal degeneration (a primary tauopathy), as well as those with AD [6].

Similar to blood and CSF, tau PET (both first- and second generation tracers) can distinguish AD from controls with high sensitivity and specificity. Whilst there is some promise for a small number of the second-generation tracers (e.g. [18F]PI-2620 and [18F]APN-1607) in identifying the presence of tau in those with PSP [7–9], a specific tracer for the 4-repeat (or 3-repeat) tauopathies remains to be developed.

No TDP-43 PET tracer has been developed as of yet, and blood and CSF measures of TDP-43 and phospho-TDP-43 that have been developed have been disappointing in their ability to detect pathological TDP-43 pathology in vivo [10]. One recent paper highlighted the development of an RT-QuIC method for detecting TDP-43 but the results from this have not yet been reproduced [11]. Perhaps the most promising biomarker for TDP-43-related disease is the measurement of *de novo* peptides in CSF resulting from the inclusion of so-called cryptic exons in transcripts due to loss of TDP-43 function [12]. Initial studies have identified a number of these peptides including HDGFL2, which seems to be present at higher concentrations in those with TDP-43 pathology [13^▪▪^,^14^^▪▪^].

FUS remains an elusive pathological entity, with no specific biomarkers yet identified. However, it is a rare cause of FTD, perhaps accounting for only around 5% of all cases.

One exception to the above in the FTD spectrum is *C9orf72-*related disease where studies have shown that the toxic dipeptide repeat (DPR) proteins produced by non-ATG translation of mutant *C9orf72* transcripts are present in the brain preceding the onset of TDP-43 pathology (Fig. 1) [15]. One of these DPRs, poly(GP) can be readily measured in CSF [16], with a more sensitive trial-ready assay on the Simoa platform having been recently developed [17]. Other DPRs have been more difficult to measure, with less clear differences between *C9orf72* expansion carriers and controls, although recent studies have managed to measure both poly(GR) and poly(GA) [18].

### Neuronal dysfunction

At some point in the disease process, abnormal deposition of tau, TDP-43 or FUS leads to neuronal dysfunction. Loss of function may precede neurodegeneration (neuronal loss) and is potentially measurable using a number of biomarkers. [18F]FDG-PET measures hypometabolism and has been shown to be abnormal many years prior to symptom onset in both *GRN* and *C9orf72* mutation carriers (even in the absence of grey matter atrophy) [19–21]. Newer methods of potentially measuring early neuronal dysfunction include arterial spin labelling MRI which shows hypoperfusion [22] and [11C]UCB-J-PET which shows decreased synaptic density [23] in the presymptomatic period.

### Neurodegeneration

Neuronal loss follows dysfunction, and this has been measured most commonly using structural T1 MRI with studies showing focal changes in the medial temporal lobes in *MAPT* mutation carriers and the insula in *GRN* mutation carriers up to 15 years prior to the estimated onset of symptoms and in the thalamus and posterior cortical regions up to 25 years prior to onset in *C9orf72* mutation carriers [24]. White matter changes can often be seen earlier using diffusion tensor imaging, with newer targeted sequences such as NODDI potentially showing changes even prior to this [25].

More recently, neurofilament light chain (NfL) has been identified as a nonspecific marker of neurodegeneration in multiple neurological disorders. In FTD, studies have so far shown increases in concentrations relatively late in the presymptomatic period and it is unclear at present whether these changes are detectable prior to grey matter atrophy [26,27].

## PRODROMAL STAGE

The prodromal stage indicates the onset of symptoms although these may be subtle at first and are often very hard to identify, particularly during a time when anxiety and depression are present [28,29^▪^]. The two main clinical rating scales used in FTD are the Clinical Dementia Rating scale plus the National Alzheimer's Coordinating Centre Frontotemporal Lobar Degeneration module (CDR plus NACC FTLD) and the FTD Rating Scale (FRS). The CDR plus NACC FTLD provides both a sum of boxes score and a global score, with the latter being used recently to more objectively place people into different disease stages, i.e. asymptomatic (score of 0), prodromal (score of 0.5) or fully symptomatic (score of 1 – mild, 2 – moderate and 3 – severe disease). A recent study comparing the CDR plus NACC FTLD with the FRS showed they were strongly correlated and both could sensitively measure longitudinal change over time when fully symptomatic, but that relatively minimal change occurred on both scales during the prodromal stage in a 1 year period [30]. The study also highlighted that the wide phenotypic spectrum of FTD was not represented in these two scales; although behaviour, language and cognition were measured, neuropsychiatric and motor features were missing. More recent work has tried to rectify this by adding in neuropsychiatric [29^▪^] and motor [31^▪^] components to the CDR plus NACC FTLD. Such work will be important in the further understanding of the concept of mild cognitive +/- behavioural +/- motor impairment (MCBMI) as a way of better describing the prodromal stage of FTD ([32^▪▪^]; Tables 1 and 2, Fig. 1).

Cognitive deficits are measurable using neuropsychological tasks in the prodromal stage and recent work has shown that the domains involved are relatively specific to the different genetic groups, e.g. early deficits of episodic memory and language are seen in prodromal *MAPT* mutation carriers [33,34], although executive function and social cognition deficits are commonly seen across the genetic groups [35]. Recently, a cognitive composite (the GENFI-Cog) unique to each genetic group has been developed for use in the prodromal period decreasing the sample size required for trials [36^▪▪^].

## PHENOCONVERSION

As people meet the diagnostic criteria for bvFTD, PPA etc. they are said to ‘phenoconvert’. However this time is not always clear cut (Table 2), and it can be difficult to predict when it will occur. Measures of ‘proximity’ to symptom onset have been investigated in recent years. As mentioned above NfL seems to change relatively late in the presymptomatic period and one study from the FPI has shown that a raised NfL at baseline predicts with good sensitivity and specificity that individuals will convert to having overt symptoms over the next couple of years [37]. A recent paper has shown that regional grey matter atrophy on MRI can also predict an increase in score on the CDR plus NACC-FTLD over time [38].

## NOVEL AREAS OF RESEARCH

Disease progression modelling has been used across different diseases to identify the timeline of biomarker changes. In FTD, models such as Subtype and Stage Inference (SuStaIn) have shown not only the changing patterns of grey matter atrophy over the presymptomatic period but have also identified specific subgroups with different trajectories [39]. A more recent study using Bayesian modelling on multimodal biomarker data from the FPI has identified a distinct temporal ordering of clinical, cognitive, MRI and NfL changes across the three main genetic groups [40^▪▪^]. Models such as these will be important for the design of future therapeutic trials for FTD, guiding the selection of outcome measures and enrolment criteria, with the potential to more accurately stage individuals.

The advances in digital and wearable technology over recent years may also be beneficial for improving the understanding of the presymptomatic stage of FTD. In particular, it may allow a more diverse population of people to enter studies through remote testing at home. Measurement of activity using actigraphy monitors such as Fitbits may allow the detection of important changes that predict symptom onset e.g. apathy has been identified as an indicator of cognitive decline in presymptomatic carriers [41], and increased physical activity may be associated with a slower disease progression [42]. Other technological advances that could prove useful in the presymptomatic period to assess cognition include indirect monitoring of phone metadata, app-based digital cognitive assessments, eye tracking assessments [43,44] and automated speech analysis. Similarly, motor deficits suggestive of ALS may be measurable with surface electromyography (EMG) [45] or muscle ultrasound [46], with upper limb function tasks and gait assessment [47] potentially measuring features of atypical parkinsonism.

## CONCLUSION

Whilst there are now more studies investigating the presymptomatic period of genetic forms of FTD there is still much to be learned. The work so far sheds light on the key phases of the presymptomatic period including the preclinical and prodromal stages, but further work is required to understand the complex interplay between clinical, cognitive, imaging and fluid biomarkers, in order to better dissect individual disease trajectories and therefore identify the optimal timepoint for treatments to be given. At present, many of the clinical trials are targeted at the symptomatic stages of the disease but a shift towards targeting the presymptomatic stage will be required if the drugs are to be able to prevent the onset of disease. There are a number of promising avenues for research that will be useful in the continued effort to find a cure for FTD but it is clear that this will require a global effort that includes individuals living with FTD, their families and loved ones, academics, clinicians, patient advocacy groups and pharmaceutical companies working together to help find effective treatments for this disease.

## Acknowledgements

*We would like to thank the GENFI Participant Engagement Board, particularly Jane Parker, for providing comments and review of the paper.*

### Financial support and sponsorship

*J.D.R. has received funding from an MRC Clinician Scientist Fellowship (MR/M008525/1), the NIHR Rare Disease Translational Research Collaboration (BRC149/NS/MH) and a Miriam Marks Brain Research UK Senior Fellowship. This work was also supported by the MRC UK GENFI grant (MR/M023664/1), the Bluefield Project and the JPND GENFI-PROX grant (2019-02248).*

### Conflicts of interest

*J.D.R. has provided consultancy or been on a medical advisory board for Alector, Aviado Bio, Arkuda Therapeutics, Wave Life Sciences, Novartis and Prevail Therapeutics.*

## REFERENCES AND RECOMMENDED READING

Papers of particular interest, published within the annual period of review, have been highlighted as:

▪ of special interest
▪▪ of outstanding interest
